# Supplementary material for: Olive Leaves and Hibiscus Flowers Extracts-Based Preparation Protect Brain from Oxidative Stress-Induced Injury
Source: Antioxidants (Basel). 2020 Sep 1;9(9):806. doi: 10.3390/antiox9090806 (PMC7555463; doi:10.3390/antiox9090806)
Supplement: Supplementary file 1 [file antioxidants-09-00806-s001.pdf]

Olive leaves and hibiscus flowers extracts-based preparation protect brain from oxidative stress-induced injury

Chiaino Elda[1], Matteo Micucci[2], Sandro Cosconati[3], Ettore Novellino[4], Roberta Budriesi[2], Alberto Chiarini[2] and Maria Frosini[1]*

Supporting Information

Figure S1. Effects of PRES on oxidative stress-induced cytotoxicity in SH-SY5Y cells. Phase-contrast microscope images: these highlighted a marked reduction in cell number accompanied by morphological changes (rounded bodies, shorter neurites, and smaller cell size, grade 2-3*) upon H_2O_2 treatment, which were gradually reverted with increasing PRES concentration. Controls (CTRL) represent untreated cells. Each photograph was representative of three independent observations (scale bar 75 μm).

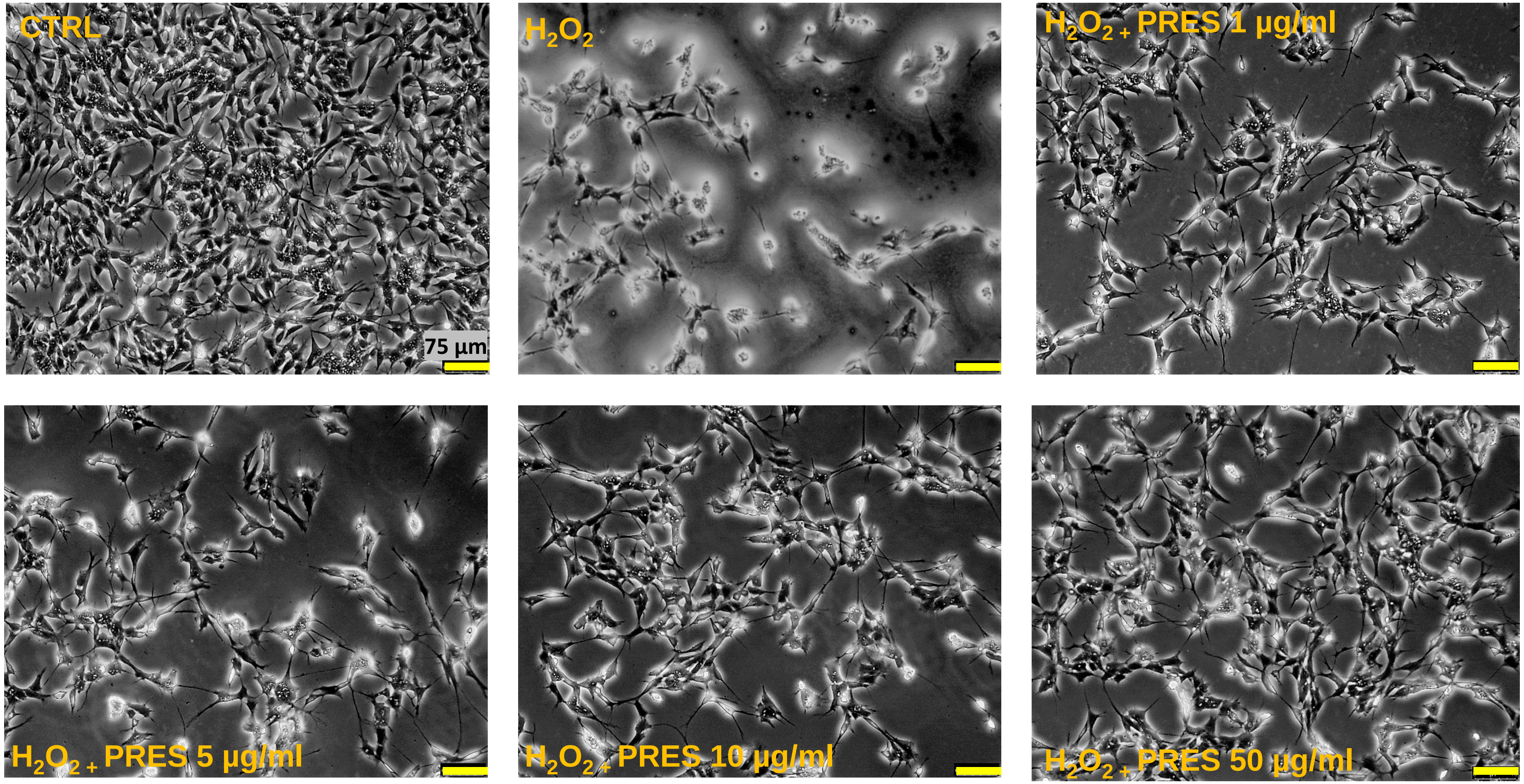

*According to the USP 28 (United States Pharmacopeia edition 2005) grade scale for assessment of the cytotoxic potential of tested materials: grade 0 - no reactivity (discrete intracytoplasmic granules, no cell lysis); grade 1 - slight reactivity (no more than 20% of the cells are round, loosely attached and without intracytoplasmic granules; occasional lysed cells are present); grade 2 - mild reactivity (no more than 50 % of the cells are round and devoid of intracytoplasmic granules, no extensive cell lysis and empty areas between cells); grade 3 - moderate (up to 70% of cells are rounded or lysed); grade 4 - severe (nearly complete destruction of the cells)[13, 16].
